# A Patient and Public Engagement Project to Inform Dementia Care in a UK Hospital Trust

**DOI:** 10.1111/hex.70024

**Published:** 2024-09-10

**Authors:** Rachel K. Marrow, Camille Cronin, Victor Ashby, Thomas Currid, Marie Alexander

**Affiliations:** ^1^ School of Health and Social Care University of Essex Wivenhoe Park Colchester UK; ^2^ School of Health and Social Care University of Essex Elmer Approach Southend‐on‐Sea Essex; ^3^ East Suffolk and North Essex Foundation Trust (ESNEFT) Turner Road Colchester Essex UK

**Keywords:** case study research, dementia, Public Patient Involvement and Engagement, service improvement, stakeholders

## Abstract

**Introduction:**

The increasing prevalence of dementia in the United Kingdom presents significant challenges for healthcare, with projections estimating over a million affected individuals by 2025, costing the NHS £6.3 billion annually. Hospital admissions among dementia patients are common, occupying about 25% of UK hospital beds and leading to prolonged stays and diminished health outcomes**.**

**Method:**

This paper presents the opening stages, part of a larger project where Patient and Public Involvement and Engagement (PPIE) was employed to understand and navigate what it means for hospital care for a person living with dementia. To understand hospital care for dementia patients, focus groups were conducted through dementia cafés in Essex and Suffolk from February to July 2023 engaging patients, carers and family members.

**Results:**

Recognised processes for reporting PPIE and thematic analysis were used and identified six themes and 21 subthemes regarding hospital care: individualised care, role of carers, basic care, interpersonal communication, information sharing and staffing.

**Discussion:**

The results from the PPIE will be used to inform and work with stakeholders through the next phases of the project, which involves examining care processes in the hospital, identifying touchpoints and evaluating these areas. The project continues to be informed by stakeholders including people living with dementia, carers and staff. Additionally, the results may inform other service providers for care enhancements, processes and delivery.

**Conclusion:**

Moving forward, the study emphasises the importance of building collaborative relationships with stakeholders involved in dementia care. Additionally, it provides insights to focus areas that are fundamental for acute care organisations when delivering care to people living with dementia. By incorporating insights from PPIE, this project seeks to identify inequalities in dementia care services, improve hospital care for people living with dementia, fostering a more inclusive and supportive healthcare environment.

**Patient or Public Contribution:**

The purpose of the study was to explore the most important issues around hospital care for people with dementia, their carers and families. Persons living with dementia, carers and family members were involved in the study through community dementia cafés and recruited to take part in focus groups to discuss hospital care for patients with dementia. The design of questions and materials for the discussions was developed through consultation with the university department's service user lead and dementia specialist and reviewed by a service user member living with dementia. A pilot focus group was conducted with a group of carers. We worked with the dementia café managers to coordinate recruitment and a suitable environment to run the focus groups.

**Trial Registration:**

Not applicable.

## 1 Introduction

The significance of safe and person‐centred care for people with dementia in hospital settings gained prominence following the UK publication of the Mid Staffordshire NHS Foundation Trust Public Inquiry [1]. The World Health Organisation [2] adopted a ‘Global action plan on the public health response to dementia 2017–2025’ whereby the world's nations have acknowledged the challenges globally for people with dementia and their families. Subsequently, several studies have explored dementia in the acute hospital setting [3, 4, 5, 6, 7, 8].

Many people over 70 years admitted to acute hospitals have dementia, delirium or both and this poses a significant threat to the health of hospitalised older adults with cognitive impairment [9]. Given the increasing concern and impact of longer hospital stays and poorer outcomes for patients with cognitive impairment [10, 11] there is a need to review and evaluate current care provision in acute hospital settings for people admitted to hospital with dementia.

## 2 Background

The number of people living with dementia in the United Kingdom is increasing and is expected to reach over a million by 2025 with a projected annual cost to the NHS of £6.3 billion [8]. Figures suggest that 25% of people in hospital beds in the United Kingdom are likely to have dementia, putting a strain on acute services [12], and in a London study, 56.5% of people over 65 with a dementia diagnosis were found to have had at least one hospital admission over a 12‐month period [13]. The Alzheimer's Society [14] reported a 27% increase in hospital admissions for people with dementia between 2015 and 2019 and predicts this trend will continue.

Patients with dementia have been shown to have longer stays than those without dementia and are more likely to experience delayed discharge [12, 15]. Evidence shows people with dementia are also likely to experience reduced health outcomes following acute care in hospital settings and further complications with delirium [16, 17, 18]. Consequently, people with dementia are increasingly frequent consumers of healthcare services, with their care often being complex and presenting significant challenges for NHS resources.

Such pressure on hospital services may be relieved through prevention of admission through additional community care [13] and increasing bed capacity in care homes to reduce the length of hospital stays [19]. However, it is also crucial that acute hospital care quality is optimised to ensure detrimental health outcomes are minimised and hospital stays are as short as possible.

The term ‘fundamentals of care’ defined by Kitson et al. [20] includes multiple aspects of basic care comprising self‐care, and environmental and physiological factors, which if not met, can lead to poor care outcomes. The Fundamental of Care Framework [21] emphasises person‐centred care, which includes three core elements required for delivering effective quality care: quality relationship between care provider and recipient, integration of physical, psychosocial and relational needs, and a supportive context for care at policy and systems levels.

Patient and Public Involvement and Engagement (PPIE) is very much integral in health and social care research and should be included in areas of service improvement. There is general agreement for a more patient‐focused input with stakeholders using evidence to inform research and underpin improvements in services [22]. Therefore, when research relates to a specific population, such as persons living with dementia, it is important that representatives from the population are included in the co‐design of research that affects their lives [23].

Initial stakeholder consultation with healthcare professionals from a local hospital Trust in 2022 highlighted a range of patient care issues related to the fundamentals of care for patients living with dementia in the acute hospital setting. Examples provided included continence issues, managing patients' pain, disorientation, decreasing independence and bed pressures. The hospital reported observing staff becoming risk averse, a phenomenon showing how safety can be prioritised over person‐centred care [24]. With increasing levels of reduced resources, staff, higher dependency levels and complex needs, patients are encouraged to stay in bed. Whilst this may reduce falls, there are increased levels of deconditioning and reduced independence in patients with dementia. Physical function becomes reduced due to inactivity, increased use of incontinence pads, and significant levels of dehydration and poor nutrition [6]. The consultation highlighted other factors including staffing pressures and stigma towards dementia in staff and public.

This led to a local partnership project between the university and local acute hospital trust with the purpose of evaluating current dementia care provision. The project adopted a case study design informed by a participatory approach, in which the knowledge and experiences of service users are used in partnership with those of staff and stakeholders [25]. Within the United Kingdom, considerable effort has been focused on developing an infrastructure to operationalise the policy commitment to PPIE [26, 27] to embed patient and public involvement engagement into their work. This participatory element, presented in this report, was to ensure that the project focused on areas of dementia care that are most relevant to the service users who will ultimately be affected by the quality of care received in the acute hospital settings.

## 3 Method

The aim of this paper is to present the participatory element of a larger partnership project, carried out between February and September 2023. This used a participatory approach to explore the views and opinions of people living with dementia and their family carers in the community regarding care in the acute hospital setting.

### 3.1 Focus Groups

Dementia cafés (sometimes known as memory cafés) are informal meeting groups that provide social spaces, activities, information and support for people affected by dementia. Dementia cafés were selected as appropriate locations for the focus groups as they provided access to people living with dementia and carers in the community, and a neutral and comfortable environment for people to attend focus groups. The senior research officer and research officer attended several dementia cafés in the area. An important part of the process was to develop an understanding of how the cafés work and support the community. Each café was visited three times: First to meet organisers and people attending the cafés and to plan the focus group; second to recruit for the focus group and provide information and consent forms; and finally, to carry out the focus groups, which were held between 26/4/2023 and 26/6/2023 (see Table 1).

**Table 1:** Summary of dementia café focus groups and participants.

		Carers		Persons living with dementia		
Date	Area	Women	Men	Women	Men	Total
24/04/2023	[Essex]	2	4	0	0	6
26/04/2023	[Essex]^a^	7	6	1	3	17
09/05/2023	[Essex]	7	2	0	0	9
12/05/2023	[Suffolk]	2	1	1	0	4
22/05/2023	[Essex]	1	0	4	0	5
25/05/2023	[Essex]	1	0	1	0	2
06/06/2023	[Suffolk]	2	2	1	0	5
26/06/2023	[Suffolk]	5	1	0	0	6
		27	16	8	3	54

^a^ Group was split into two focus groups.

The focus group format was prepared by the research team, which includes dementia nurse specialists and a family carer, in consultation with a service user living with dementia. The first focus group acted as a pilot, after which one question was reworded following feedback.

### 3.2 Participants

**Figure 1:**
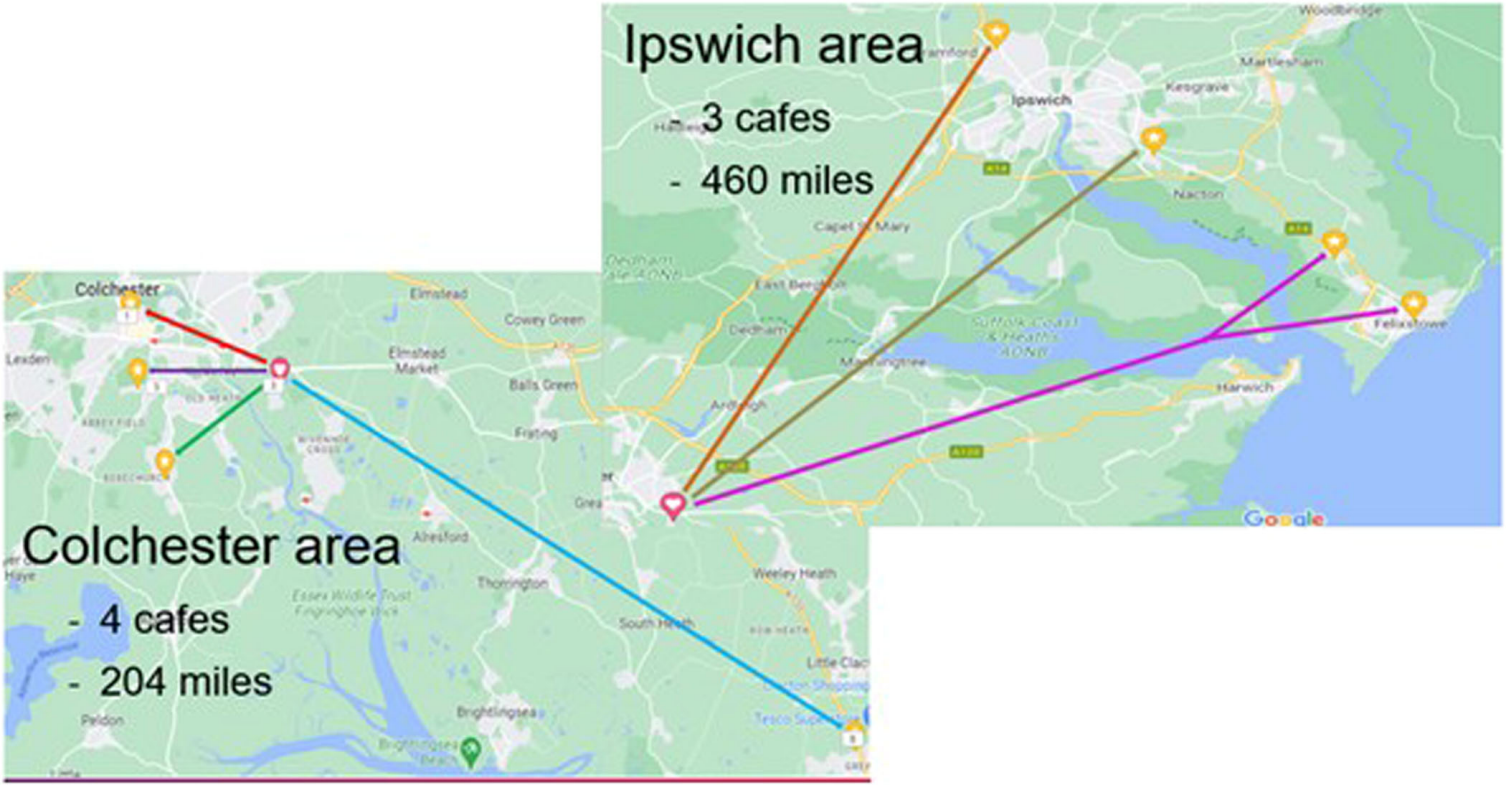
Map of areas travelled to for dementia café focus groups.

There were 54 participants in nine focus groups at seven different dementia cafés (Table 1) in Essex and Suffolk (Figure 1). Eleven participants were living with dementia and the remainder were carers. One carer was working as a professional carer for someone with dementia while the rest were family carers (sometimes referred to as informal carers). The carer was either currently involved in the care of a family member living with dementia (*n* = 39) or had previously been the primary carer of a family member with dementia who had since died (*n* = 3). Experiences of participants varied including a mix of direct experience of acute hospital care whilst their family members were living with dementia; and views, opinions and concerns about possible future hospital care of the person whom they are looking after.

### 3.3 Ethical Approval

The study was approved by the University of Essex Research Ethics Committee (March 2023) ETH2223‐0647. The main ethical consideration was to ensure participants felt relaxed and comfortable, so focus groups were held in a separate room at the same venue and time as the dementia cafés, except for one, where the group took place in a familiar community setting. A safe and private space was available at each venue should anyone require it. Mental capacity assessments were carried out just before focus groups for participants with dementia, to ensure they had the capacity to understand their role in the focus group and its purpose.

### 3.4 Data Collection

The focus group format was prepared in consultation with nurse specialists and tested using a pilot group. Focus groups had a semi‐structured format centred around four questions relating to hospital care, written on individual cards. Two questions involved asking attendees to orally share answers with the group in turn, and two required a response to be written on a ‘post‐it note’ and the group was asked if anyone wished to expand on their responses (Table 2). Attendees were encouraged to take turns to read out the questions to the group to increase interaction and involvement. At each focus group, one researcher facilitated the discussion, and the other observed and took notes on the discussion. Attendees were offered a notebook to provide an opportunity to consider questions in their own time and completed notebooks were brought back to the cafés.

**Table 2:** Focus groups questions.

Icebreaker (selected from five questions)	What is your favourite hobby/place/biscuit/ice cream or song?
Question 1	On a post‐it note, please write a word or phrase that comes to mind when you think of going to hospital. If you like, you can draw a picture.
Question 2	Imagine you or someone you know who has dementia is in hospital. What does it feel like?
Question 3	What can the hospitals do to make hospital stay as comfortable as possible for someone living with dementia? Please write your answers on the post‐it notes.
Question 4	As you know we are doing a project with your local hospital to try and improve hospital care for people with dementia. What would you like us to focus on?

### 3.5 Data Analysis

The results of the focus groups were analysed using a thematic analytical approach to identify themes and subthemes [28]. This involved familiarisation of the notes by reading them several times and highlighting comments of interest, known as codes. The next stage was to look for patterns across the codes and to summarise these by themes and subthemes, involving the research team and subsequently checking that themes reflected all focus group discussions.

## 4 Results

The analysis produced six themes and 21 subthemes (Table 3). This section presents the narrative data from each theme: individualised care, the role of carers, basic care, interpersonal communication, information sharing, staffing issues and their subthemes with narrative excerpts from people living with dementia and carers.

**Table 3:** Themes and subthemes.

Theme	Subthemes
Individualised care	Respecting the elderly and persons living with dementia
	Dementia is different for each person
	Respecting diversity
	Patient wellbeing
Role of carers	Fears
	Challenges
	Carers should be involved
	Carers should have extended visiting
Basic care	Dignity
	Cleanliness
	Nutrition
	Safety
	Mobility
Interpersonal communication	Communication from hospitals to carers/families
	Staff/patient communication
	Understanding patient behaviour
Information sharing	Sharing information
Staffing issues	Not enough time
	More training is needed
	Not enough staff
	Staff culture and attitudes

### 4.1 Theme 1. Individualised Care

This theme relates to care being personalised to the patient living with dementia, with a positive relationship between healthcare professionals and patients feeling valued and cared for.

#### 4.1.1 Respecting the Elderly and Persons Living With Dementia

This subtheme reflects concerns that patients with dementia can be underestimated. For example, an attendee with vascular dementia said:

When the hospital staff are aware the patient has dementia, they assume they don't know anything.

(Person living with dementia)

Another contributor thought his wife's care was less prioritised due to her dementia, saying:

My wife is treated differently because she has dementia and there is a decreased priority.

(Carer)

#### 4.1.2 Dementia is Different for Each Person

This reflects a common discussion point that dementia affects each person in a different way and care needs should be adjusted accordingly. A participant with Alzheimer's said:

Dementia affects people differently, so they need to be cared for differently.

(Person living with dementia)

Presentation of dementia also varies due to the different stages of dementia, which can be challenging for those in the early stages. A carer said:

My wife who is living with dementia looks at other people with dementia as strange, especially those who are further down the spectrum.

(Carer)

#### 4.1.3 Respecting Diversity

Several factors were discussed relating to diversity, including culture, religion and language barriers. For example, a contributor from the Bangladeshi community said:

Bangladeshi women are often more isolated because they rarely leave the family home. This would lead to even more confusion and stress if they had to go to hospital because of cultural shock.

(Carer)

One attendee felt that people with early onset dementia may require a different approach to care, suggesting:

Everyone is different and there are younger adults with dementia who need to be approached differently at the hospital.

(Carer)

#### 4.1.4 Patient Wellbeing

There were concerns that the effects of being in hospital on mental health can sometimes be overlooked by staff. A carer, whose husband has dementia and was in hospital at the time of the focus group, spoke of his difficulties, saying

I wish the hospital would understand—he has COPD, Parkinson's, and diabetes. He's very closed off. He's very down.

(Carer)

Some of the focus group discussions turned to ideas to improve patient wellbeing, for example, a carer suggested.

Nurses should greet patients sometimes, especially when they are idle. A conversation could cheer them up (Carer).

(Carer)

### 4.2 Theme 2. The Role of Carers

The theme of ‘The role of carers’ reflects the strong view, held by carers and those with dementia, that carers are extremely important in the care of persons living with dementia in acute settings and should be regarded as the experts in their care.

#### 4.2.1 Carer Fears

Several carers were worried about what would happen should their loved ones require hospital care. One carer expressed a general fear of hospital care, saying:

Fear of the unknown. Fear of the promises.

(Carer)

The close relationships between family carers and long‐term partners were discussed in several focus groups. Effective care in the home had often been difficult to achieve and carers felt they were in a unique position to understand their partner's needs. Some were concerned they would lose control of care if their loved one needed to go to hospital:

You have no control.

(Carer)

#### 4.2.2 Carer Challenges

An array of issues around the practical challenges of caring for a person living with dementia and carer availability. Although most focus group attendees either had carers or were carers, a contributor raised concerns that not everyone was in this position:

AG wondered what would happen to people visiting the hospital without carers.

(Carer)

#### 4.2.3 Carers Should Be Involved

A commonly expressed view by carers was that they could help with the care of patients with dementia. Suggestions included helping with communication, at mealtimes, and with care plans. Another frequent suggestion was that carer assistance could reduce the workload of healthcare professionals.

Allow at least one close family to be with them and help them and then give the nurses more time to do other things.

(Person living with dementia)

#### 4.2.4 Carers Should Have Extended Visiting

A common opinion was that carers should have additional visiting times. A service user with dementia who was cared for by her husband said:

Having my husband with me makes me less nervous.

(Person living with dementia)

### 4.3 Theme 3. Basic Care

This theme relates to the basics of hospital care for people living with dementia.

#### 4.3.1 Dignity

This relates to respect and dignity, for example, the overuse of incontinence pads rather than helping patients to use bathrooms. Others were more general, for example, a contributor living with dementia stated she would want hospital staff to:

Have time and intuition to support my daily needs with dignity.

(Person living with dementia)

#### 4.3.2 Cleanliness

A small number of carers had concerns about cleanliness, for example, a carer whose husband was in hospital at the time of the focus group stated:

I visit him from 10am to 4 pm. Sometimes he needs a wash. His hands are always crusty with food. I normally feed him.

(Carer)

#### 4.3.3 Nutrition

This subtheme highlights the importance of positive mealtime experiences to encourage people living with dementia to eat well. Some were concerned due to staff pressures, patients who require more time to eat are sometimes missed by staff:

L stated that housekeeping brings out the food tray, but it is almost like a drop off. They would then return in 20 minutes and wonder why the patient didn't eat their food.

(Carer)

#### 4.3.4 Safety and Supervision

There were conversations around security on wards and patient safety, for example, a carer who attended Accident and Emergency had concerns for a patient with dementia who was on his own:

There was a gentleman there with dementia, and no one was with him. No staff were checking up on him and the ambulance staff were not able to keep up. I helped him. I don't know why he was alone.

Carer

#### 4.3.5 Mobility

This reflects concerns that once admitted to hospital, patients with dementia are encouraged to stay in bed and this can lead to reduced mobility when discharged. One contributor shared her experience regarding her brother, saying:

D told a story about her brother who had to stay in bed when in the hospital so that he ended up in a home because he couldn't walk any more. She was scared that this would happen to her husband.

(Carer)

### 4.4 Theme 4. Interpersonal Communication

Much of the interpersonal communication theme relates to person‐centred care and best practices.

#### 4.4.1 Communication From Hospitals to Carer/Families

This mainly related to the experiences of family and carers who felt communication from the hospital had been insufficient or confusing. For example, a carer reported there was no notification of his wife being moved from the hospital to a care home.

I phoned up a couple of days later and was told that she was not there on the ward. They had sent her to a care home. No one notified me.

(Carer)

#### 4.4.2 Patient‐to‐Staff and Staff‐to‐Patient Communication

Challenges do arise in communicating with a patient with dementia, both from the patient's and staff's perspectives. For example, patients with dementia may become confused by their surroundings, and not remember instructions. A service user reported her mother became upset and confused when she needed help, but staff were not available.

Nobody had time to take her to the toilet. Her anxiety was raised, she didn't understand how to call for help. Mum was so very upset.

(Carer)

#### 4.4.3 Understanding Patient Behaviour

Various examples were given of how aspects of hospital care could affect behaviour, which could then be challenging to understand. For example, a carer stated that agitation in patients with dementia can easily occur in a hospital environment, saying,

Time taken for the nurses to assess can be too long and agitation happens quickly.

(Carer)

Another carer said.

If they are not taking things on board, they might be scared and not say anything.

(Carer)

### 4.5 Theme 5. Information Sharing

This theme relates to how medical records and other information are shared within the hospital setting. One concern was that information was not always passed between hospital staff, with one carer explaining he had to repeatedly explain to staff that his wife had dementia:

I said that she had dementia and the one thing they did not do is pass the information on to the next shift.

(Carer)

Another aspect of this theme was that information recorded in patient notes could be shared in such a way that could hold detrimental consequences for future patient care: An example is carers who felt their husbands were labelled as aggressive due to instances of agitated behaviour.

Two carers said that their husbands had been labelled as aggressive and it affected which care homes would accept them.

(Focus group notes)

Some comments, however, related to experiences of information being shared in a positive way, for example, one carer said a 'This is Me booklet' had been helpful during her husband's stay in hospital.

They made my husband a booklet (this is me), with pictures and information about him.

(carer)

### 4.6 Theme 6. Staffing Issues

There was a general positive regard and gratitude towards the hospital staff in the PPIE focus group discussions, however, there were concerns about various staffing issues.

#### 4.6.1 Insufficient Staff

Staffing numbers were viewed as too low. One carer said:

The hospital I went to had 2 nurses on the ward. The lack of staff at the hospital, could mean everyone's needs cannot be met.

(Carer)

#### 4.6.2 More Training is Needed

Some people thought there were issues with training, especially around understanding care needs for patients with dementia, for example, one person said:

Nurses seem not to be trained to recognise dementia and this is important.

(Person living with dementia)

#### 4.6.3 Insufficient Time

People reported staff shortages and high workloads. One service user, a retired nurse living with dementia, thought this affected the level of compassion in care provided:

M said she didn't think people have the time for compassion in hospital. When you have loads of people who all have different ideas its difficult…The job of a nurse is hard when you have lots of patients wanting different things.

(Person living with dementia)

There were concerns that staff pressures affected the amount of time spent with patients, with one carer saying:

They (acute staff) don't have enough time to listen.

(Carer)

#### 4.6.4 Staff Culture and Attitudes

Some people thought work culture and attitudes towards patients with dementia affected care. One person said:

P said when she was in hospital she was dismissed by the staff when she told them she needed something for her diabetes. She said she was dismissed as a person, like they attributed everything she did to her dementia.

(Person living with dementia)

## 5 Discussion

The purpose of this project was to understand the opinions and experiences of people living with dementia and their carers regarding hospital care, enabling the project to focus on factors of most concern to service users, their families and carers. The focus groups took place in familiar surroundings to the participants at several dementia cafés in Essex and Suffolk. The six themes arising from the focus groups: *individualised care, the role of carers, basic care, interpersonal communication, information sharing and staffing issues* will be discussed.

### 5.1 Individualised Care

The ‘individualised care’ theme relates to discussions on the importance of treating people as individuals, with both people living with dementia and carers noting that dementia affects people very differently and at different stages. Some contributors with dementia felt they were not treated as individuals due to their dementia, others felt individuality can be overlooked especially in people who are elderly, and others highlighted differences between cultures.

High‐quality, individualised care for people living with dementia can be achieved through a person‐centred approach [29]. Person‐centred care puts the patient at the core of their care and is built upon a strong care provider to care recipient relationship, requiring trust to be at the relationship's nucleus [21]. Focus group discussions reflected all elements of the care received, but in this theme, the extent of care was personalised and the quality of relationships between healthcare providers, patients and carers was key. Person‐centred care can improve patient wellbeing, quality of life and reduce agitation [30].

There are constant challenges to providing person‐centred care. Within a busy, task‐focused acute setting, staff persistently face challenges related to time and pressures when caring for persons living with dementia needing complex treatment [7]. Caring priorities are normally centred around diagnostic procedures, close monitoring and treatment in acute settings [7, 29, 31]. Although training in dementia awareness can help improve the standard of dementia care [5], it has also been found that time pressures can prevent the long‐term benefits of training [32]. Consequently, quality person‐centred care for people living with dementia should be prioritised, yet at times it is difficult to attain.

### 5.2 The Role of Carers

One common view across the focus groups was the importance of involving family carers in delivering quality hospital care. Carers can provide comfort and reassurance to the patient and help with some elements of care, whilst benefitting themselves by feeling included. Carers were sometimes fearful that hospital staff would not understand patients' needs, and that the hospital environment could be distressing and confusing.

The idea that informal carers can provide significant support to people living with dementia who are in hospital reflects an established view [33]. Informal carer involvement has been found to improve clinical and psychosocial outcomes, such as treatment adherence, symptom reduction, treatment satisfaction and reduce the need for rehospitalization [34]. Evidence suggests that the absence of a family carer may have a detrimental effect on patient wellbeing [35, 36].

However, there are challenges when involving carers with one study suggesting that carer expectations can sometimes be unrealistic [37], and a recent study suggested carers did not always possess the necessary knowledge to communicate effectively with hospital staff [38]. Furthermore, carers are not always available or often do not wish to be involved [35], while challenges include working with exhausted family carers, dealing with family conflicts, safeguarding and differing opinions about the patient's care [39].

The subtheme ‘carer fears’ illustrates how family carers can be concerned about the prospect of their family member having to be hospitalised. Although not necessarily drawing on personal experiences, research has shown that hospitalisation can be difficult for carers, and can make them feel vulnerable and on edge [40]. It is also the case that many family carers have their own care needs, for example, they may have a long‐term condition or dementia themselves or have difficulty coping [41]. One suggested approach is to view the carer and patient together as a ‘unit of care’ [36].

### 5.3 Basic Care

The theme ‘basic care’ reflects discussion around basic elements of hospital care, mostly related to experiences or stories the participants had heard where care was viewed as inadequate. For example, where patients were unsupervised, and not given enough opportunities to get out of bed or enough help to eat. An important element of this theme was dignity, with some participants feeling this was not always prioritised. Some participants felt reduced basic care quality was linked with staff shortages, reflecting similar findings in previous research [42]. A measure shown to help mitigate working pressures seen in staff is to involve carers, volunteers and support groups in providing basic care needs [39]. In this way, this theme is related to the theme ‘the role of the carers’.

Research suggests there can be limited understanding and knowledge of dementia in hospital staff [41]. Dementia training has been shown to improve the standard of care of patients with dementia, although the level of improved practices can be dependent on supportive management and a positive working culture [43, 44]. There are also challenges to quality care when patient safety is prioritised, resulting in patients’ dignity and wellbeing being overlooked in favour of measures aimed at preventing falls and injuries, which can lead to pressure areas or deconditioning [45]. Thus, a balance between immediate care needs and safety, and longer‐term consequences can be difficult to achieve.

### 5.4 Interpersonal Communication

The theme ‘interpersonal communication’ is an interrelated theme that crosses over ‘the role of the carer’, ‘staffing issues’ and ‘sharing information’. Within the theme itself, there are two elements of communication in the hospital: communication between staff and patients, and from the hospital to carers.

Interpersonal communication for a patient living with dementia in an acute care setting can be difficult and is echoed in a recent literature review [39]. Assessing a patient with dementia can often be difficult for staff, from initial assessment in the acute phase to ongoing care, evaluating pain, and meeting nutritional and hydration needs [46]. People living with dementia may not cooperate with healthcare staff because of their difficulty in understanding treatment rationale, which can be further compounded by different cultural understandings of care [39].

Quality individualised care can be improved by optimising healthcare staff's ability to pick up on a patient's non‐verbal communication, which may be difficult to interpret, but can make the patient feel safe and reassured when correctly understood [43]. Also, the implementation of initiatives, such as ‘This is me’, [47] can help with patient preferences, although this is not mandatory or widely used across health and social care settings.

The use of relational approaches has been seen to be the most beneficial approach to the provision of effective communicative care for people living with dementia. This starts with establishing rapport with a patient and is easily achieved by offering simple gestures of help and using appropriate communication skills [48].

Involving family carers in patient care can help staff communicate effectively with patients and identify how patients express pain, provide accurate accounts of the patient's medical history and reassure the patient if necessary [39].

### 5.5 Information Sharing

Some of the focus groups included participants who had experienced difficulties with communication between departments and systems at the hospital. For example, patients’ dementia not being communicated between staff and patients being labelled as aggressive. Positive information sharing included the use of ‘This is Me’ [47].

It has previously been found that hospital systems do not communicate effectively with one another, and sometimes when a patient is moved to another ward, patient information does not always follow to the new ward [49].

One way of communicating when a patient has dementia is by using visual identifiers. Various systems are used in the United Kingdom, including a forget‐me‐not sticker from the Alzheimer's Society. In a study of UK hospitals, mostly positive views of visual identifiers were found, although negative issues included inconsistencies of use, lack of training and ethical concerns of consent [50].

Digby et al. [45] reported a general stigma surrounding dementia, and patients with dementia being stigmatised, sometimes due to specific behaviour. For example, a patient could be deemed difficult due to task‐focused nursing care having initiated agitated or aggressive behaviour. It is important that a distinction is made between reactive aggression, usually as a result of a lack of understanding in the patient that leads to them rejecting care, and proactive aggression due to psychopathic symptoms, delusions or hallucinations [51]. Failure to distinguish between the two types of aggression can lead to incorrect treatment and inappropriate labelling of patients as aggressive [51].

### 5.6 Staffing Issues

The theme ‘staffing issues’ represents perceived issues such as insufficient staff in the hospitals and high levels of sickness. Additionally, there were concerns that staff were not trained sufficiently to understand the care needs of patients with dementia, and that sometimes the lack of understanding and time can lead to a reduction in the quality of care provided. The predominant view across the focus groups was that staff are well meaning but are facing challenges that make quality care more difficult to attain.

The association between staffing levels and patient or system outcomes is not new [39]. Appropriate staffing ensures an effective balance between the needs of patients and the ability of nurses to perform care tasks, but higher staffing workloads have been shown to be associated with poor patient outcomes [42]. Care omissions may be a result of acute staff experiencing time pressures that affect observation and attention to the complex needs of patients [39].

High workloads affect staff in various ways, for example, leading to burnout and emotional exhaustion [42]. It has been suggested that high workloads can lead to staff seeing patients with dementia as an inconvenience, as their care can take longer [24]. A lack of knowledge about dementia and work pressures can lead to low staff morale [41].

Additional training in dementia can help alleviate staffing issues, for example, improving communication and increasing empathy in staff [43] as well as increasing positive staff attitudes towards patients with dementia. Thus, reskilling of staff can help the hospital alleviate elements of the working pressures seen with acute staffing [42]. However, a lack of resources can be a barrier to training being successful, both in terms of training attendance and implementing new skills, and the role of supportive management has been shown to be pivotal in facilitating successful positive change [44].

### 5.7 Strengths

The PPIE element of the project used the voices of people living with dementia and carers from local dementia cafés to inform a larger partnership project. Voices of people living with dementia are pivotal to the project and key to ensuring that the results are representative. Collaboration with dementia cafés enabled relationships with communities in the local area to be built. The research team continues to foster these relationships throughout the duration of the project to ensure it remains informed and with the stakeholders involved in dementia care.

The six themes: *individualised care, the role of carers, basic care, interpersonal communication, information sharing and staffing issues* are being embedded into three key steering groups; a dementia steering group, stakeholder group, and hospital dementia and delirium group, thus informing future dementia services.

### 5.8 Limitations

By utilising dementia cafés, contributors were restricted to those who attended the cafés, which not all people living with dementia, or their carers are able to or wish to do. Due to the nature of dementia and the dementia café format, it was difficult to recruit many carers or people with dementia with direct experience of acute hospital care or who could recall their experiences. Therefore, views expressed were drawn from a mix of direct and indirect experiences, and concerns for the future.

Another limitation could be the perceived lack of diversity in the focus groups, which were largely white and fluent in English, reflecting the local population at the time.

Additionally, despite measures taken by the research team to make participants as comfortable and as included as possible some participants may have felt unsure about sharing their views and experiences in a focus group environment.

## 6 Conclusion

By using PPIE, six themes have been identified that reflect the voices of people who experience dementia. These themes will inform the work of the project and the wider partnership approach, enabling the researchers and stakeholders to use an evidence‐informed participatory approach to ensure that people affected by dementia are informing services in the acute hospital setting.

## Author Contributions

**Camille Cronin:** methodology, supervision, funding acquisition, validation, formal analysis, writing–original draft, writing–review and editing, conceptualisation, project administration, resources. **Rachel K. Marrow:** writing–original draft, writing–review and editing, formal analysis, methodology, investigation, validation, resources, project administration, data curation. **Victor Ashby:** investigation, methodology, resources, formal analysis, writing–review and editing, project administration. **Thomas Currid:** writing–review and editing, conceptualisation, methodology, supervision, formal analysis, validation. **Marie Alexander:** writing–review and editing, supervision.

## Ethics Statement

The study was approved by the University of Essex Research Ethics Committee (March 2023) ETH2223‐0647.

## Consent

All participants provided written informed consent before participating.

## Conflicts of Interest

The authors declare no conflicts of interest.

## Data Availability

Data will not be available online.
